# Manganese-Titanium Mixed Ion Sieves for the Selective Adsorption of Lithium Ions from an Artificial Salt Lake Brine

**DOI:** 10.3390/ma16114190

**Published:** 2023-06-05

**Authors:** Yaxuan Ding, Nguyen Thi Hong Nhung, Jiahao An, Hao Chen, Lianying Liao, Chunlin He, Xinpeng Wang, Toyohisa Fujita

**Affiliations:** School of Resources, Environment and Materials, Guangxi University, Nanning 530004, China; 2115391100@st.gxu.edu.cn (Y.D.); hnhung_2912@yahoo.com (N.T.H.N.); anjiahao@st.gxu.edu.cn (J.A.); hchen1996@st.gxu.edu.cn (H.C.); liaolianying2022@163.com (L.L.); helink@gxu.edu.cn (C.H.); wangxinpeng@gxu.edu.cn (X.W.)

**Keywords:** lithium-ion sieve, salt lake brine, DL-malic acid, ion exchange, lithium selectivity

## Abstract

Lithium recovery is imperative to accommodate the increase in lithium demand. Salt lake brine contains a large amount of lithium and is one of the most important sources of lithium metal. In this study, Li_2_CO_3_, MnO_2_, and TiO_2_ particles were mixed, and the precursor of a manganese–titanium mixed ion sieve (M-T-LIS) was prepared by a high-temperature solid-phase method. M-T-LISs were obtained by DL-malic acid pickling. The adsorption experiment results noted single-layer chemical adsorption and maximum lithium adsorption of 32.32 mg/g. From the Brunauer–Emmett–Teller and scanning electron microscopy results, the M-T-LIS provided adsorption sites after DL-malic acid pickling. In addition, X-ray photoelectron spectroscopy and Fourier transform infrared results showed the ion exchange mechanism of the M-T-LIS adsorption. From the results of the Li^+^ desorption experiment and recoverability experiment, DL-malic acid was used to desorb Li^+^ from the M-T-LIS with a desorption rate of more than 90%. During the fifth cycle, the Li^+^ adsorption capacity of the M-T-LIS was more than 20 mg/g (25.90 mg/g), and the recovery efficiency was higher than 80% (81.42%). According to the selectivity experiment, the M-T-LIS had good selectivity for Li^+^ (adsorption capacity of 25.85 mg/g in the artificial salt lake brine), which indicates its good application potential.

## 1. Introduction

Based on the continuous development of new energy, lithium is the core element of power battery production, which has become a strategic resource for global competition and has been widely used in the manufacture of electric vehicles [1,2], biomedicine [3], nuclear fusion reaction [4], and ceramic [5]. Due to the rapid development of electric energy products and the global popularity of lithium batteries in recent years, the demand for lithium has been continuously increasing [6]. As projected, lithium would be requiring 498 kilo tons of Li_2_CO_3_ in 2025 [7]. However, lithium recycling is no longer sufficient to meet lithium demand. According to Swain, the rate of global lithium recycling in 2017 was less than 1% [8]. Therefore, it is necessary to achieve stable and sustainable lithium recovery from nature. Lithium resources exist in lithium ores, seawater, and salt lake brine [9,10,11]. Lithium in salt lake brine accounts for approximately 80% of the total lithium resources [2], making it one of the most important lithium sources. However, the high magnesium–lithium and sodium–lithium ratio in most salt lakes makes lithium extraction challenging [12,13]. Therefore, the effective separation between lithium and magnesium, sodium, is a significant research subject.

The methods for extracting lithium metal from salt lake brine include precipitation [14], solvent extraction [15], ion exchange [16], electrodialysis [17], and membrane separation [18]. Although precipitation is a simple method for recovering lithium from salt lake brine, the impurity ions in the brine affect the purity of lithium and its recovery efficiency [2]. In addition, extracting lithium from salt lake brine using extractants pollute the water [19]. The electrochemical method has the advantage of high efficiency. However, economic benefits and energy consumption are among the challenges for lithium recovery by the electrochemical method [20]. The adsorption materials have advantages in economy and environmental protection due to their simple preparation process, low energy consumption, high recovery efficiency, and good selectivity.

As a method for adsorbing and recovering lithium ions, a lithium-ion sieve (LIS) has the advantages of high adsorption capacity and selectivity for lithium and is often used for extracting lithium from salt lake brine. LISs are divided into lithium manganese oxide [21] and lithium titanium oxide [22] types. However, they are restricted in the application due to the reusability and adsorption performance of lithium manganese oxide are not prominent, and the low adsorption efficiency of lithium titanium oxides. Liu et al. [23] found that the LIS precursor Li_1.6_Mn_1.6_O_4_ prepared by sol-gel hydrothermal synthesis and two-step heat treatment had lithium adsorption of only 10.05 mg/g in salt lake brine. Li et al. [24] obtained a mixed LIS based on TiO_2_ with a solution pH of 12. However, a low adsorption capacity of only 14 mg/g was obtained. Lin et al. [25] designed Li/Al LDHs with a layered structure to study the adsorption performance of Li^+^ in interlayer water and high Mg^2+^/Li+ ratio brine. Li/Al LDHs had low adsorption capacity due to the influence of interlayer water molecules and the explanation of the adsorption mechanism of layered adsorption still remained a challenge for practical application [26]. In addition, doping other metals into LISs could improve their lithium adsorption [27,28]. However, the preparation process used was complex and the adsorption performance of the material was not prominent. Therefore, it was necessary to obtain LISs with a simple preparation process and high adsorption capacity for the recovery of lithium ions from salt lake brine.

The pickling treatment of LIS precursor had a crucial influence on the performance of LIS. The HCl solution was commonly used as the eluent of the LIS precursor [21,28,29,30]. However, the volatility of HCl, its harmfulness to physical health, and its pollution of the environment limited its wide application. Organic acids had the characteristics of no pollution and low corrosion to equipment, which could effectively replace HCl as the pickling medium for lithium-ion sieves. However, there were few relevant reports. Wang et al. [31] used citric acid (C_6_H_8_O_7_) to obtain a titanium-ion sieve with significant selectivity and characterization and significantly reduced titanium loss. Citric acid (C_6_H_8_O_7_), tartaric acid (C_4_H_6_O_6_), and DL-malic acid (C_4_H_6_O_5_) had a similar chemical structure, and they had the advantage of being non-toxic and pollution-free, and it had good selectivity for lithium ions, which could effectively elute lithium ions from LISs.

In this study, to investigate the influence of mixing manganese and titanium on lithium adsorption, the mixture of Li_2_CO_3_, MnO_2_, and TiO_2_ was sintered by solid-phase sintering to prepare new material, referred to as manganese–titanium mixed with LIS (M-T-LIS) precursor. Evaluate the effects of citric acid, tartaric acid, and DL-malic acid on the adsorption of M-T-LIS. The adsorption performance and optimum parameters of lithium were evaluated by the adsorption experiments. The structure and pore morphology of the M-T-LIS were analyzed by X-ray diffraction (XRD), scanning electron microscopy (SEM), and Brunauer–Emmett–Teller (BET) methods. The chemical mechanism of the lithium adsorption by the M-T-LIS was analyzed by X-ray photoelectron spectroscopy (XPS) and Fourier transform infrared (FT-IR). In addition, DL-malic acid was used as the desorption agent of Li^+^. The desorption effect of DL-malic acid on Li^+^ was studied, and the optimal desorption conditions were investigated. Further, the selectivity of M-T-LIS to Li^+^ was analyzed using a simulated brine, which explained the effect of M-T-LIS on the lithium extraction from salt lake brine.

## 2. Materials and Methods

### 2.1. Materials

**Figure 1:**
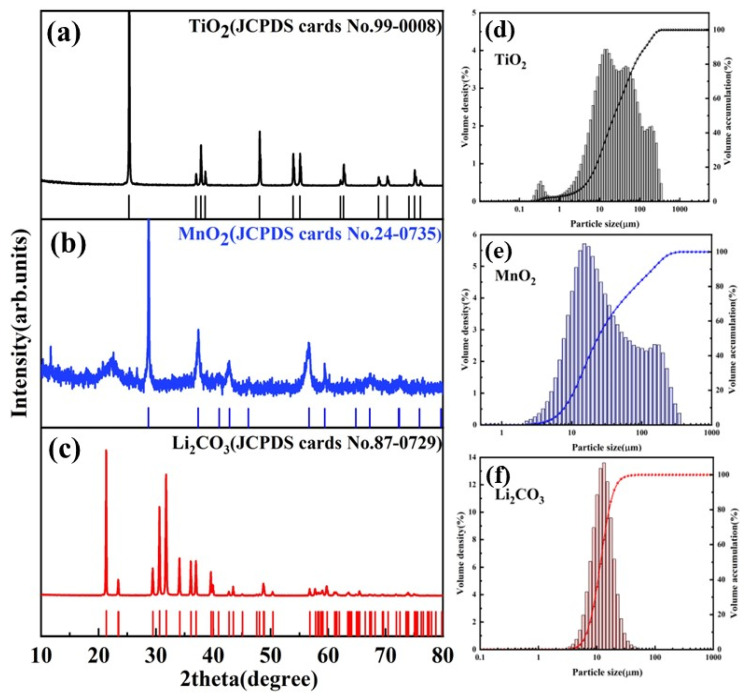
(**a**–**c**) XRD spectra of TiO_2_, MnO_2_, and Li_2_CO_3_, respectively. (**d**–**f**) Particle size distribution of TiO_2_, MnO_2_, and Li_2_CO_3_, respectively.

Lithium carbonate (Li_2_CO_3_, AR, 99.5%) and manganese dioxide (MnO_2_, AR, 85%) particles were purchased from Shanghai McLean Biochemical Technology Co., Ltd. (China). Titanium dioxide (TiO_2_, AR, >99.0%), citric acid (C_6_H_8_O_7_, AR, more than 99.5%), and lithium chloride (LiCl·H_2_O, AR, >97.0%) particles were obtained from Guangdong Guanghua Technology Co., Ltd. (Guangzhou, China). The XRD diffraction pattern of TiO_2_, MnO_2_, and Li_2_CO_3_ particles are shown in Figure 1a–c. The particle size distribution of TiO_2_, MnO_2_, and Li_2_CO_3_ is shown in Figure 1d–f. The mean size of TiO_2_, MnO_2_, and Li_2_CO_3_ particles are 22.97, 23.37, and 11.65 µm, respectively. DL-malic acid (C_4_H_6_O_5_, AR, >99.0%) was purchased from Shanghai Aladdin Biochemical Technology Co., Ltd. (Shanghai, China). Tartaric acid (C_4_H_6_O_6_, AR, more than 99.5%) was purchased from China National Pharmaceutical Group Chemical Reagent Co., Ltd. (Shanghai, China). Deionized water (18.2 MΩ·cm) was used for the solution preparation and testing.

The artificial salt lake brine configured in this study was based on the ion species and concentration of the concentrated saltwater in West Taijinar brine of China. The artificial salt lake brine was prepared with Li^+^ (LiCl: 582 mg/L), K^+^ (KCl: 17,416 mg/L), Ca^2+^ (CaCl_2_: 165 mg/L), Na^+^ (NaCl: 13,770 mg/L), and Mg^2+^ (MgCl_2_: 48,467 mg/L) to configure the artificial salt lake brine obtained from Guangdong Guanghua Technology Co., Ltd., Shenzhen, China.

### 2.2. Preparation of M-T-LIS

The Li_2_CO_3_, MnO_2_, and TiO_2_ particles with the Li:Mn:Ti molar ratio of 2:1:1 was added to the mortar and then ground for 30 min for thorough mixing to obtain a mixture of Li_2_CO_3_, MnO_2_, and TiO_2_ particles. The mixture was placed in a tubular furnace and heated at 298 K to 873 K at a heating rate of 5 K/min under an N_2_ atmosphere for 240 min and then naturally cooled to room temperature to obtain the M-T-LIS precursor. The prepared M-T-LIS precursor was immersed in 0.3 mol/L (S/L = 4 g/L) citric acid, tartaric acid, and DL-malic acid and then stirred for 1440 min to obtain the M-T-LIS. The M-T-LIS was washed with deionized water until a neutral pH is obtained and subjected to suction filtration. The filtered powder was placed in an oven at 323 K for drying for 480 min until the moisture in the powder completely evaporates.

### 2.3. Material Characterization

The characteristics of LTM were studied by thermogravimetric analysis–differential scanning calorimetry (TGA-DSC, Netzsch, STA 449 F3 Jupiter, Selb, Germany). XRD (Smart lab, Rigaku, Tokyo, Japan) was conducted using CuKα at 2*θ* of 10°–80°. The particle size of the TiO_2_, MnO_2_, and Li_2_CO_3_ particles was analyzed by a laser particle size analyzer (LA-960A, Horiba, Kyoto, Japan). The concentration of the Li^+^ ions in the LiCl solution before and after adsorption was measured by an atomic absorption spectrophotometer (AAS, AA-7000, Shimadzu, Japan). The specific surface area and pore size distribution of the M-T-LIS and its precursor were obtained using a specific surface area and pore size analyzer (TriStar II 3020, Micromeritics, Norcross, GA, USA). The specific surface area and average pore diameter of the sample were obtained by the BET method. The surface charge of the M-T-LIS was measured by a zeta potential analyzer (Nanobrook Omni, Brookhaven, Holtsville, NY, USA). A carbon–sulfur analyzer (Leco, CS844, St. Joseph, MO, USA) was used to analyze the mass percentage of carbon in the M-T-LIS precursor before and after its preparation and pickling. Field-emission SEM (TESCAN MIRA LMS, Brno, Czech Republic) was used to analyze the particle morphology of the M-T-LIS and its precursor. XPS (Thermo Fisher Scientific ESCALAB 250Xi, Waltham, MA, USA) was used to characterize the M-T-LIS precursor before and after pickling, and adsorption by M-T-LIS. FT-IR (Shimadzu IRTracer-100, Shimadzu, Kyoto, Japan) was used to characterize the changes in the functional groups before and after pickling the M-T-LIS and the adsorbed powders in the range of 400–4400 cm^−1^. The ion concentration of the simulated brine before and after adsorption was measured by an inductively coupled plasma atomic emission spectrometer (ICP-OES, Shimadzu, Japan).

#### 2.3.1. Lithium Adsorptive Experiments

LiCl·H_2_O and deionized water were used to prepare a lithium chloride solution with a theoretical concentration of 100 mg/L. The actual concentration was determined by AAS. The prepared M-T-LIS was added to the prepared LiCl solution, and the ratio of the adsorption solution to M-T-LIS was 500 mL/g. The sample bottle containing the M-T-LIS and LiCl solution was placed on a vibrator with an oscillation frequency of 140 rpm to vibrate until the solution reached equilibrium or the adsorption process was completed. The adsorbed solution was first filtered by suction. After removing the solution after filtration, the solubility of Li^+^ in the solution was measured after adsorption with AAS, and the adsorption capacity [32] of the M-T-LIS was calculated using Equation (1):

(1) $$Q_{e}=\frac{\left(C_{0}-C_{e}\right)V}{m}$$

where *Q_e_* (mg/g) is the Li^+^ ion adsorption capacity; *C*_0_ (mg/L) is the initial concentrations of Li^+^; *C_e_* (mg/L) is the equilibrium of Li^+^ ion; *V* (L) is the volume of the resulting solution; and *m* (g) is the weight of the adsorbents.

The solution pH significantly influences Li^+^ adsorption, and the research of Wang et al. [33] showed that Li^+^ can be well adsorbed in an alkaline buffer solution. Therefore, the influence of pH value on the adsorption effect was investigated. Therefore, M-T-LIS was added to 2 mol/L NaOH to adjust the pH of the prepared LiCl solution (adjusted pH: neutral, 8, 10, 12, 12.5), and the optimal pH was determined by calculating the adsorption capacity.

The optimal pH was determined. Subsequently, the M-T-LIS was added to the LiCl solution adjusted to the optimal pH, and the influence of the adsorption time (stirring time of 10–840 min) on the adsorption effect was studied. In addition, the influence of the temperature on the adsorption experiment was investigated after determining the optimal pH and time. Finally, the M-T-LIS was added to the LiCl solution at different temperatures (303–328 K) to select the best adsorption temperature.

#### 2.3.2. Equilibrium Isotherm Studies

Adsorption isotherms are considered to provide key data to describe the interaction between the adsorbate and adsorbent. LiCl solutions with different initial concentrations (10–100 mg/L) were prepared. The LiCl solutions were then adjusted with varying concentrations to the best conditions for studying the adsorption isotherms. The M-T-LIS was placed into the LiCl solutions of different concentrations with the liquid–solid ratio maintained at 500 mL/g. After the adsorption, the concentration under the equilibrium condition with the supernatant was measured. Langmuir (Equation (2)) and Freundlich [34] (Equation (3)) adsorption isotherms were used for the fitting of the experiment.

(2) $$Q_{t}=\frac{q_{m}K_{L}C_{e}}{\left(1+K_{L}C_{e}\right)}$$

*Q_e_* = *K_F_C_e_^n^* (3)

where *q_m_* (mg/g) represents the maximal quantity of Li^+^ ion carried by per unit gram LIS; *C_e_* (mg/L) is equilibrium solution concentration; *K_L_* (L/g), *K_F_* (mg^1−n^ g^−1^L^n^), and *n* are the constants of the Langmuir model or Freundlich equation associated with the compatibility of the adsorbent binding site.

#### 2.3.3. Adsorption Kinetics Studies

In this study, pseudo-first-order kinetics (Equation (4)) and pseudo-second-order kinetics (Equation (5)) models [35] were used for the linear fitting analysis of the Li^+^ ion adsorption kinetics:

In (*Q_e_* − *Q_t_*) = In*Q_e_* − *K*_1_*t* (4)

(5) $$\frac{t}{Q_{t}}=\frac{1}{K_{2}Q_{e}}+\frac{t}{Q_{e}}$$

where *Q_e_* (mg/g) is the adsorption capacity of Li^+^ ion on the adsorbent; *Q_t_* (mg/g) is the adsorption capacity of Li^+^ ion on the adsorbent at time *t* (min); and *K*_1_ (min^−1^) and *K*_2_ (g/mg/min) are the pseudo-first-order and pseudo-second-order rate constants, respectively.

The M-T-LIS was added to the prepared LiCl solution. The liquid–solid ratio was maintained at 500 mL/g, and the oscillation treatment was conducted with an oscillation frequency of 140 rpm. At various times (5–840 min), the samples were extracted, and the adsorption capacity of M-T-LIS was investigated.

#### 2.3.4. Recyclability of the Adsorbents

The desorption and recyclability of the adsorbents are necessary for the study of M-T-LIS adsorption. As the DL-malic acid readily reacts and complexes with lithium [36], DL-malic acid was selected as the desorption solution to study the effect of the desorption concentration and time. DL-malic acid with concentrations of 0.00–0.30 mol/L was prepared for the desorption experiment. The M-T-LIS adsorbed under the optimal adsorption conditions was added to the configured DL-malic acid with the fixed liquid–solid ratio of 500 mL/g and placed in the oscillator with the oscillation frequency of 140 rpm for the desorption experiment. After determining the optimal concentration of the DL-malic acid, the effect of the experiment on the desorption was studied. The M-T-LIS was added into the DL-malic acid with the fixed liquid–solid ratio of 500 mL/g, which was under the optimal adsorption conditions, and then placed in an oscillator with an oscillation frequency of 140 rpm for the desorption experiments. The samples were removed at different times (120–720 min), and the desorption capacity (Equation (6)) and desorption efficiency (Equation (7)) were calculated:

(6) $$Q_{d}=C_{d}\times \frac{V}{m}$$

(7) $$E_{d}=\frac{q_{d}}{q_{e}}\times 100\%$$

where *C_d_* is the concentration of Li^+^ in the solution after desorption, *V* (L) is the volume of the Li^+^ solution, *m* (g) is the mass of the adsorbent, *Q_d_* (mg/g) is the desorption capacity, and *E_d_* (%) is the desorption efficiency.

To study the recyclability of the M-T-LIS, the Li^+^ concentration was 100 mg/L, the DL-malic acid was selected as the desorption solution, and the ratio of the solution to solid is 500 mL/g. The cycle experiment was conducted under optimal adsorption and desorption conditions.

#### 2.3.5. Selective Experiment

To study the selectivity of the M-T-LIS in salt lake brine, this study simulated the environment of a salt lake brine and conducted selectivity experiments. First, the artificial salt lake brine was adjusted to the optimal pH using 2 mol/L NaOH solution. The M-T-LIS was then added to the prepared solution and placed into the oscillator with an oscillation frequency of 140 rpm. The temperature was adjusted to the optimal temperature. The initial concentration of the solution and concentration after adsorption was measured by ICP-OES. The distribution coefficient (*K_d_*, mL/g, Equation (8)) and separation factor of the Li^+^ selectivity (*α*, Equation (9)) were calculated to evaluate the selective recovery capacity of the M-T-LIS for Li^+^:

(8) $$K_{i}=\frac{q_{Me}}{C_{Me}}\times 1000$$

(9) $$\alpha =\frac{K_{d}^{Li}}{K_{d}^{Me}}$$

where *Me* represents Li^+^ and other competing ions; and *q_Me_* (mg/g) and *C_Me_* (mg/L) represent the adsorption capacity and concentration of Li^+^ and other competing ions, respectively.

## 3. Results and Discussion

### 3.1. TGA-DSC and XRD Analysis

**Figure 2:**
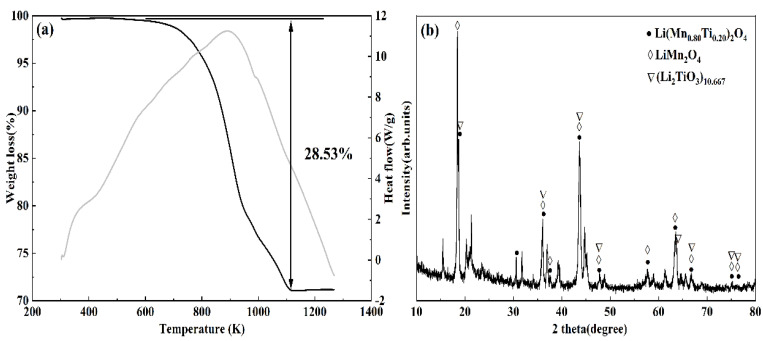
(**a**) TGA results of the M-T-LIS precursor at 302–1268 K. (**b**) XRD analysis of the M-T-LIS.

At the beginning of the TGA curve (Figure 2a), a gradual weight loss occurred at 302–600 K owing to the water evaporation in the material. According to the TGA-DSC curve, a rapid weight loss of 28.53% occurred when the temperature was heated from 600 K to 1118 K. The DSC curve exhibits the same results. The reduction of the carbon mass percentage before and after sintering the M-T-LIS precursor (Table 1) explains that the weight loss is caused by the decomposition of Li_2_CO_3_ [37]. The stabilization of the M-T-LIS structure and generation of more voids on the M-T-LIS surface follows the reaction in Equation (10) during pickling to increase the contact area between the pickling solution and Li^+^. Therefore, the M-T-LIS precursor was prepared at 873 K.

CO_3_^2−^+2H^+^ → CO_2_↑+H_2_O (10)

The M-T-LIS precursor was obtained by sintering the manganese, titanium, and lithium sources at high temperatures. The XRD patterns of the M-T-LIS precursor are shown in Figure 2b. After the high-temperature sintering of LMT, the M-T-LIS obtained is composed of Li(Mn_0.80_Ti_0.20_)_2_O_4_ (JCPDS cards No. 89-0124), LiMnO_4_ (JCPDS cards No. 89-0106), and (Li_2_TiO_3_)_10.667_ (JCPDS cards No. 75-1602). The peak strength indicates the high crystallinity of the M-T-LIS, and other diffraction peaks may be unreacted and bound to TiO_2_, MnO_2_, and other components. From the XRD results, M-T-LISs has a good crystallization effect, indicating the stable structure of the M-T-LIS precursor sintered at 873 K.

### 3.2. SEM and BET Analysis

**Figure 3:**
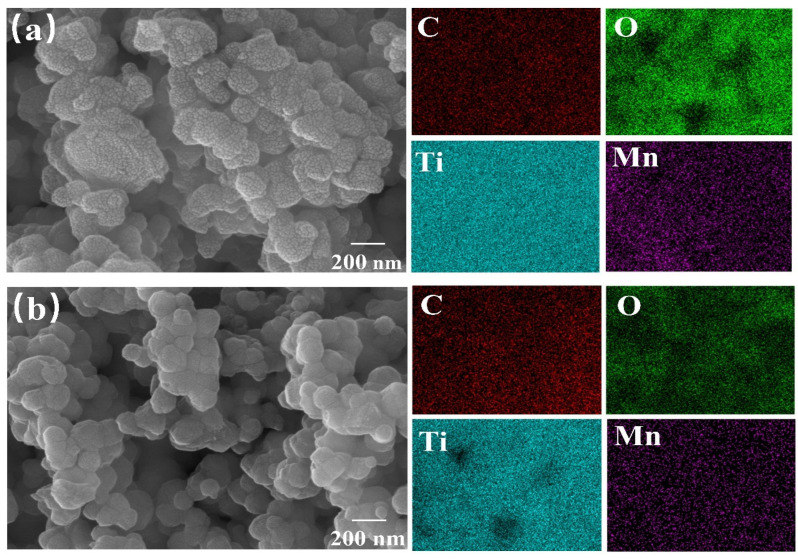
SEM images of (**a**) the M-T-LIS precursor and (**b**) the M-T-LIS after DL-malic acid pickling.

SEM was used to scan the M-T-LIS and its precursor (Figure 3). After high-temperature sintering, the Li_2_CO_3_, MnO_2_, and TiO_2_ particles are regularly agglomerated and tightly combined, and the precursor particles exhibit a rough surface (Figure 3a). After pickling, the agglomeration structure of the M-T-LIS has not changed, and its hole size increased significantly (Figure 3b). The M-T-LIS precursor was subjected to DL-malic acid pickling to obtain the M-T-LIS. The carbon mass of the M-T-LIS before and after acid pickling was measured by a carbon–sulfur analyzer (Table 1). The carbon mass decreases from 4.39 to 0.18 owing to the reaction in Equation (10), resulting in large holes, as shown in Figure 3b.

**Figure 4:**
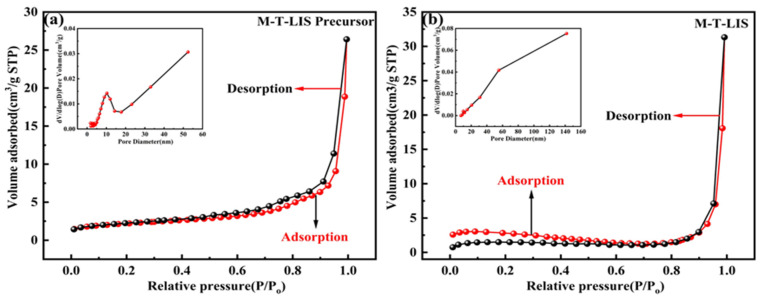
N_2_ adsorption–desorption isotherm and pore size distribution of the (**a**) M-T-LIS precursor and (**b**) M-T-LIS.

The average specific surface area, average pore size, and pore volume of the M-T-LIS increase (Table 1). As shown in Figure 4a, the M-T-LIS precursor has a type IV N_2_ adsorption–desorption isotherm, and H_3_ hysteresis loop [38] (IUPAC classification), indicating that the M-T-LIS precursor is a mesoporous material. After DL-malic acid pickling, macropore structures are formed in M-T-LIS (Figure 4b), indicating the reaction of H^+^ in the solution with CO_3_^2-^ in the M-T-LIS during pickling, thereby exposing more Li^+^ sites in the structure. In addition, the adsorbent exhibits a smooth surface after pickling, which can be ascribed to the elution of lithium compounds (Li^+^) on the M-T-LIS surface and the formation of lithium monolayer adsorption sites. These results demonstrate the good elution effect of DL-malic acid on the Li^+^ content on the surface of the M-T-LIS without affecting the stability of the structure.

### 3.3. Lithium Adsorptive Experiments

#### 3.3.1. Influence of the Initial pH

**Figure 5:**
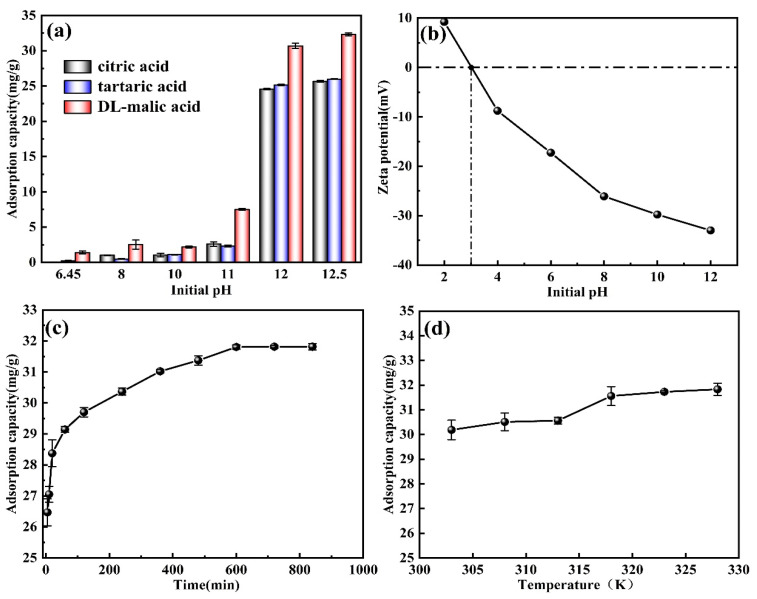
Influence of the (**a**) initial pH of M-T-LIS pickling by citric acid, tartaric acid, and DL-malic acid (*C_Li_*_+_: 100 mg/L, temperature: 303 K, time: 600 min), (**b**) zeta potential at different pH, (**c**) time (*C_Li_*_+_: 100 mg/L, temperature: 303 K, pH: 12), and (**d**) temperature (*C_Li_*_+_: 100 mg/L, time: 600 min, pH: 12) on the adsorption capacity of the M-T-LIS.

Figure 5a shows the effect of M-T-LIS pickling by citric acid, tartaric acid, DL-malic acid, and pH on the lithium adsorption by the M-T-LIS. The adsorption experiment results show that the M-T-LIS precursor has an increased adsorption capacity for Li^+^ after pickling by citric acid, tartaric acid, and DL malic acid. When the pH is equal to 12, the adsorption capacity of M-T-LIS reaches: citric acid (25.67 mg/g), tartaric acid (25.99 mg/g), DL-malic acid (32.32 mg/g). Due to the chelating performance of DL-malic acid on Li^+^ [36,39], more Li^+^ is eluted during the pickling process, providing richer adsorption sites. PH has a significant impact on the adsorption capacity of M-T-LIS. The adsorption capacity of the M-T-LIS for Li^+^ is extremely low in the pH range of 6–10 and increases sharply when the pH value is 11–12. According to the zeta potential (Figure 5b), when the pH of the solution is 2–3, the M-T-LIS has a positive surface potential but is not conducive to Li^+^ adsorption. With the increase in pH, the surface potential of the M-T-LIS becomes negative, and the potential gradually increases. When the pH reaches 12, the negative potential value reaches the maximum value, indicating that the solution is more conducive to the adsorption of Li^+^ by the M-T-LIS, which is mainly ascribed to the alkalinity of the adsorption system. Further, a large amount of OH^-^ in the solution is bound to H^+^ the M-T-LIS site [40] further promoting the adsorption of Li^+^ by the M-T-LIS. Therefore, pH considerably influences the adsorption capability of the M-T-LIS. When the initial pH of the solution is higher than 12, the lithium adsorption effect gradually increases, indicating that the adsorption has gradually reached equilibrium. This may also be attributed to Li^+^ occupying several lithium adsorption sites on the M-T-LIS surface, which makes it difficult to continue adsorption. Although increasing the OH^-^ concentration can further promote Li^+^ adsorption, this results in the wastage of the alkaline solution and an increase in environmental pollution. Thus, in this study, the optimal initial pH was set to 12. According to the change in the adsorption capacity with pH, the M-T-LIS adsorption may also be related to ion exchange.

#### 3.3.2. Influence of Time

Figure 5c shows the effect of time on the adsorption capacity. At the first 120 min, the M-T-LIS has an extremely high adsorption rate of Li^+^. In particular, the adsorption capacity of the M-T-LIS reaches 29.83 mg/g, which accounts for 93.80% of the equilibrium adsorption capacity. This can be ascribed to the large amount of Li^+^ in the solution at the beginning of the adsorption, which promoted the adsorption and occupation of the active sites on the M-T-LIS surface. As the adsorption progressed, the number of sites decreases, the adsorption rate decreases until 600 min, and the adsorption reaches equilibrium at the capacity of 31.80 mg/g.

#### 3.3.3. Influence of Temperature

The influence of temperature on the M-T-LIS was studied (Figure 5d). With the increase in temperature, the high temperature enhances the mobility of the lithium ions in the solution, thereby accelerating the diffusion of Li^+^ to the interior of the adsorbent particles [41]. The exchange efficiency of Li^+^ and H^+^ on the surface of the M-T-LIS increases [42], which gradually increases the adsorption capacity of the M-T-LIS. This suggests that the adsorption of M-T-LIS is an endothermic process. When the temperature is 318 K, the adsorption reaches equilibrium with the adsorption capacity of 31.80 mg/g. However, when the temperature exceeds 318 K, the adsorption capacity of the M-T-LIS gradually increases significantly with the increase in temperature, indicating that the adsorption reaches the equilibrium point at 318 K, which can be ascribed to the complete occupation of the Li^+^ adsorption sites on the surface of M-T-LIS. Therefore, the M-T-LIS adsorption is monolayer adsorption.

### 3.4. Adsorption Isotherm and Kinetics

**Figure 6:**
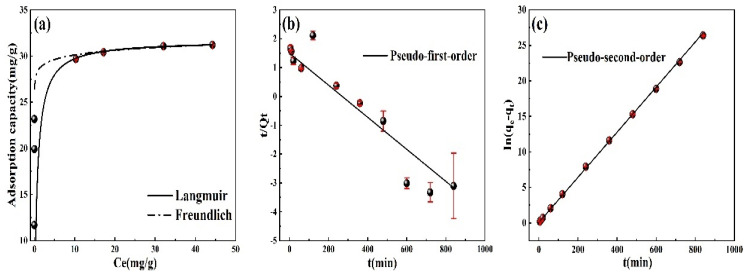
(**a**) Adsorption isotherm of the M-T-LIS. (**b**) Pseudo-first-order kinetic model of the M-T-LIS. (**c**) Pseudo-second-order kinetic model of the M-T-LIS.

Figure 6a shows the relationship between the equilibrium concentration and adsorption capacity when the M-T-LIS adsorbs Li^+^ ions. The relevant parameters after the Langmuir and Freundlich fitting are shown in Table 2. When the equilibrium concentration is less than 10 mg/L, the adsorption capacity of the M-T-LIS increases significantly. Meanwhile, when the equilibrium concentration is greater than 10 mg/L, the adsorption capacity of the M-T-LIS slowly increases until equilibrium. Combining the fitting data in Figure 6a and Table 2, the M-T-LIS conforms to the Langmuir fitting results with a high R^2^ of 0.99, demonstrating that the M-T-LIS exhibits monolayer adsorption, and the adsorption sites on the adsorbent surface are uniform. Therefore, the M-T-LIS adsorption process occurs gradually until the sites on the surface of the M-T-LIS are entirely occupied.

In this study, the pseudo-first-order and pseudo-second-order kinetic models were used for the linear fitting analysis of the Li^+^ ion adsorption kinetics (Figure 6b,c and Table 3). The results are consistent with the pseudo-second-order kinetics (R^2^ = 0.99), indicating the chemical adsorption behavior of the M-T-LIS [43]. The adsorption of the M-T-LIS occurs rapidly at the beginning and slowly increases with the increase in the adsorption time. Hence, the M-T-LIS follows single-layer chemical adsorption after acid pickling by DL-malic acid. Further, the H^+^ in the acid exchanges with the Li^+^ on the surface of the M-T-LIS precursor to obtain the M-T-LIS.

### 3.5. Lithium Desorption Experiments

**Figure 7:**
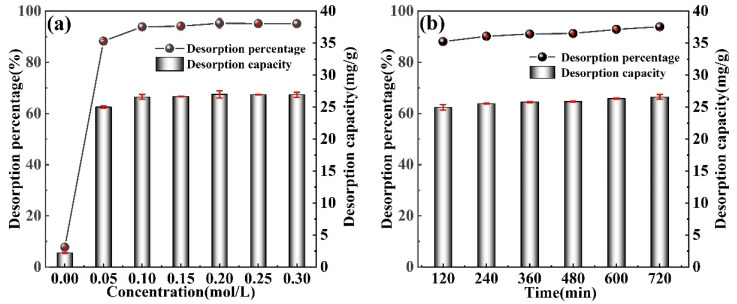
(**a**) Effect of concentration on the desorption percentage and capacity (desorption solution concentration: 0.00–0.30 mol/L, desorption time: 720 min). (**b**) Effect of time (desorption time: 120–720 min, desorption solution concentration: 0.10 mol/L) on the desorption percentage and capacity.

Figure 7a shows the effect of the desorption solution concentration (0.00–0.30 mol/L) on the desorption effect. After 600 min of desorption of the M-T-LIS with deionized water, the desorption capacity can reach 6 mg/g. When the concentration of the desorption solution is increased to 0.05 mol/L, the desorption rate will exceed 88%. When the concentration of DL-malic acid is 0.1 mol/L, the desorption rate is 93.87%. In addition, with the increase in the H^+^ concentration, the resolution rate did not significantly change, indicating the complete desorption of Li^+^ on the M-T-LIS surface. Thus, the desorption effect is prominent at the DL-malic acid concentration of 0.1 mol/L. Figure 7b shows the influence of the desorption time (120–720 min) on the desorption effect. At 120 min, the desorption rate reaches equilibrium. At 240 min, the optimal desorption rate reaches 90.16%. This suggests that the strong complexation of the malic acid with Li^+^ and monolayer chemisorption of M-T-LIS promotes the desorption of Li^+^ on the surface of the M-T-LIS when the DL-malic acid is used to desorb Li^+^. Therefore, M-T-LIS has the advantages of fast desorption speed and high desorption efficiency.

### 3.6. Selective Experiment

**Figure 8:**
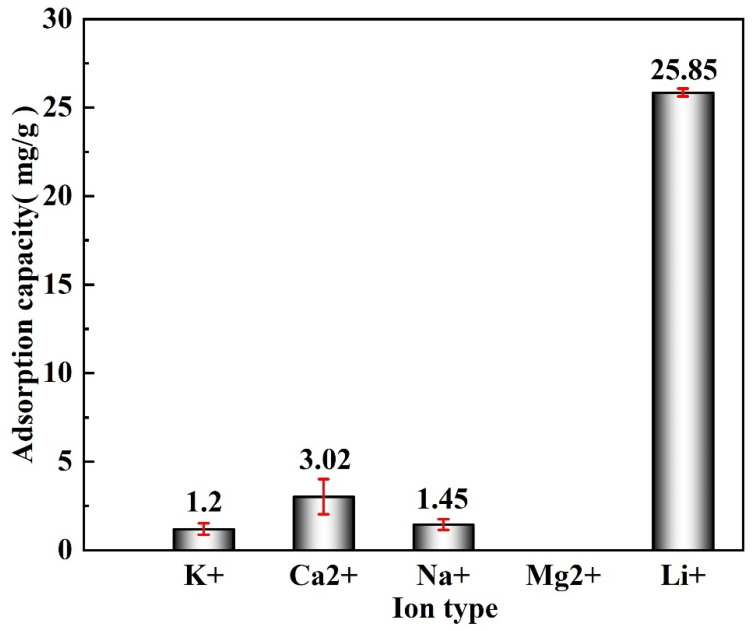
Li^+^ adsorption selectivity from the artificial salt lake brine on the M-T-LIS (pH: 12, T: 318 K, t: 600 min).

Salt lake brine has coexisting cations with similar size, and physical and chemical characteristics, such as K^+^, Ca^2+^, Na^+^, Mg^2+^, and Li^+^. Therefore, the selectivity of the M-T-LIS should be evaluated. The M-T-LIS was immersed into a simulated saline solution containing K^+^, Ca^2+^, Na^+^, Mg^2+^, and Li^+^ (S/L = 500 mL/g), the initial brine pH was adjusted to 12, and the adsorption temperature and time were controlled to 318 K and 600 min, respectively, to investigate the selectivity of the M-T-LIS. As shown in Figure 8, the highest adsorption capacity of the M-T-LIS for lithium is 25.85 mg/g. In addition, the concentration of other ions in the solution has not changed, indicating that the M-T-LIS selectively adsorbed Li^+^. The K^+^ concentration decreases to 0 when the pH value reaches 12, and the Mg^2+^ precipitates in the brine solution; however, this has minimal effects on the Li^+^ adsorption [44].

The partition coefficient (*K_d_*) and separation factor (*α*), which is the selectivity of the M-T-LIS to Li^+^_,_ was evaluated. The distribution coefficients and separation factors of the ions in the simulated brine are calculated using Equations (8) and (9), respectively, and the results are shown in Table 4. For the M-T-LIS, Li^+^ has a higher *K_d_* and *α*, indicating the high selectivity of the M-T-LIS for Li^+^ in the coexistence system of multiple ions in brine, which is mainly related to the hydration radius [45]. The hydration radius of Li^+^ is larger than that of K^+^ and Na^+^, and more binding sites are captured in the competitive reaction process. As the hydration radius of Ca^2+^ is larger than that of Na^+^, the presence of Ca^2+^ further hinders the site capture ability of Na^+^ [46]. In addition, the M-T-LIS has good Li^+^ selective adsorption capacity in natural brine with high Na^+^/Li^+^ concentrations. Because of the extremely small ion radius of Li^+^, the M-T-LIS produces a large number of Li^+^ adsorption sites after pickling, which leads to the rapid occupation of Li^+^ sites, further hindering the binding of other ions to sites.

## 4. Adsorption Mechanism

**Figure 9:**
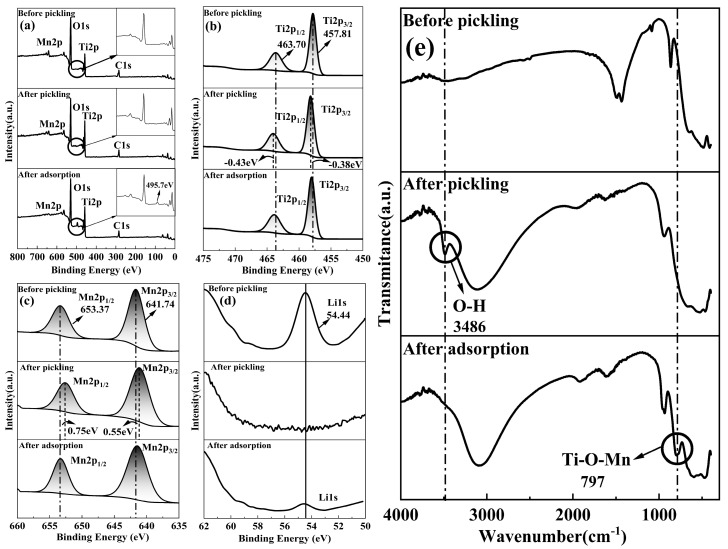
XPS characterization of the M-T-LIS before and after pickling and adsorption. (**a**) XPS survey spectra (inset is the partial enlargement) and high-resolution XPS spectra of (**b**) Ti2p, (**c**) Mn2p, and (**d**) Li1s. (**e**) FT-IR spectra for the M-T-LIS before and after pickling, and after adsorption.

The lithium ions exhibit different selective recovery mechanisms. Kim et al. [47] selectively recovered lithium ions from the brine solution through a continuous redox reaction. In addition, Nie and Bai et al. [16,48] demonstrated the selective recovery of Li^+^ from the salt lake brine through ion exchange. In this study, XPS was used to measure the chemical composition of the M-T-LIS precursor and M-T-LIS after pickling and adsorption. FT-IR was used to analyze the adsorption mechanism of the M-T-LIS before and after pickling. The movement of the characteristic peaks [49,50,51,52] of Ti2p_1/2_ (0.43 eV), Ti2p_3/2_ (0.38 eV), Mn2p_1/2_ (0.75 eV), and Mn2p_3/2_ (0.55 eV) in Figure 9b,c, the disappearance of the characteristic peak [52] of lithium (Figure 9d), and infrared spectrum (Figure 9e), the vibration peak [53,54] of O–H appears at 3500–3400 cm^−1^ indicate that Li^+^ in the M-T-LIS is replaced by H^+^. The chemical environment of Mn and Ti changes from Mn–O–Li, Ti–O–Li, and Mn–Ti–O–Li to Mn–O–H, Ti–O–H, and Mn–Ti–O–H, respectively. After the Li^+^ adsorption, the characteristic peak of Li_1s_ reappeared (Figure 9d), suggesting the successful adsorption of Li^+^. The XPS spectra in Figure 9b,c show that the characteristic peaks of Ti2p_1/2_, Ti2p_3/2_, Mn2p_1/2_, and Mn2p_3/2_ after Li^+^ adsorption are consistent with those of Ti and Mn before pickling, indicating that the chemical environment of Mn and Ti is maintained. Moreover, the infrared spectrum image shows the disappearance of the characteristic peak of O–H after Li^+^ adsorption (Figure 9e), which changes Mn–O–H, Ti–O–H, and Mn–Ti–O–H to Mn–O–Li, Ti–O–Li, and Mn–Ti–O-Li, respectively. From the spectrum after adsorption in Figure 9a, a characteristic peak is noted at 495.73 eV, which has not been reported in previous studies [52,55,56,57,58]. From the infrared spectrum image (Figure 9e) a vibration peak appears at approximately 800 cm^−1^ after pickling, which can be ascribed to the Mn entering the TiO_2_ lattice during pickling [59] and combining with Li^+^ after forming a composite with TiO_2_. The characteristic peak in Figure 9a can be related to this result.

## 5. Adsorption Performance

**Figure 10:**
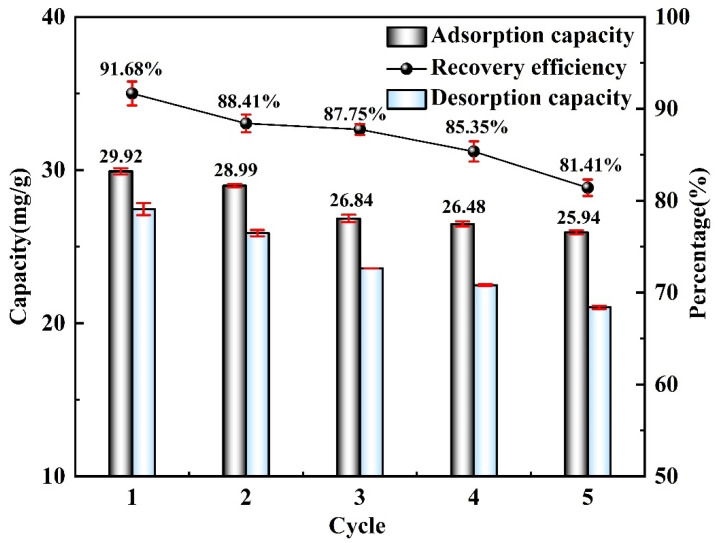
Recyclability of the M-T-LIS for Li^+^ adsorption (adsorption conditions: C_Li+_: 100 mg/L, pH: 12, temperature: 318 K, time: 600 min; desorption conditions: desorption solution concentration: 0.10 mol/L, time: 240 min).

The reusability of the M-T-LIS was investigated under repeated adsorption and desorption experiments; the results are shown in Figure 10. From the first to fifth adsorption–desorption cycles, the Li^+^ adsorption capacity of the M-T-LIS decreases from 29.92 mg/g to 25.94 mg/g, and the recovery efficiency decreases from 91.68% to 81.84%. This is attributed to the dissolution rate of the manganese and titanium ions during the adsorption–desorption process of the M-T-LIS, thereby decreasing the Li^+^ adsorption performance. In the fifth cycle, the MT-L-LIS still exhibits a high Li^+^ adsorption capacity of more than 20 mg/g and a recovery rate of more than 80%, which demonstrates the good Li^+^ adsorption–desorption performance and stability of the M-T-LIS. Moreover, these results suggest the long-term potential of the M-T-LIS for Li^+^ adsorption.

## 6. Conclusions

In this study, the M-T-LIS precursor was prepared by solid-state sintering followed by acid pickling using DL-malic acid. The adsorption experiment using the prepared M-T-LIS was performed in an artificial salt lake brine. The following results were obtained.

- Evaluated the adsorption performance of M-T-LIS precursor after organic acid treatment. After the precursor of M-T-LIS pickling by citric acid, tartaric acid, and DL-malic acid, the adsorption capacity of M-T-LIS for Li^+^ was compared under the optimal adsorption conditions: DL-malic acid 32.32 mg/g, citric acid 25.67 mg/g, and tartaric acid 25.99 mg/g. This indicated that DL-malic acid had a more significant elution performance on the M-T-LIS precursor. At the same time, the adsorption data of M-T-LIS were fitted with a first-order kinetic model and Langmuir isotherm model, and it was found that M-T-LIS belonged to single-layer chemical adsorption.
- The Li^+^ selectivity of M-T-LIS was evaluated in an artificial salt lake saline solution. The (*K_d_*) and separation factors(*α*) of the M-T-LIS for K^+^, Ca^2+^, Na^+^, and Li^+^ in an artificial salt lake brine were calculated. The results indicated that M-T-LIS had satisfactory leaching Li^+^ selectivity, making it well utilized in the study of lithium extraction from salt lake brine. By simulating the adsorption performance of M-T-LIS in actual salt lake brine, the adsorption capacity of M-T-LIS in artificial salt lake brine was 25.85 mg/g.
- The mechanism of M-T-LIS adsorbing Li^+^ ion in artificial salt lake brine was analyzed using XPS and FT-IR. XPS and FT-IR analysis indicated that M-T-LIS adsorption was carried out through ion exchange between H^+^ and Li^+^.
- The cycling performance of materials is also one of the important indicators for measuring material stability. The M-T-LIS exhibited significant Li^+^ adsorption and desorption properties after five adsorption–desorption cycles. In the fifth adsorption cycle, a high adsorption capacity of more than 20 mg/g (25.90 mg/g) was maintained, and the recovery rate of Li^+^ was 81.41%, which demonstrated the Li^+^ recovery efficiency and structural stability of M-T-LIS. Therefore, the method combining DL-malic acid and M-T-LIS to recover Li^+^ from salt lake brine had a good application prospect. This study would be used in the application of actual brine water.

## Figures and Tables

**Table 1:** BET surface area and average point size of the M-T-LIS before and after pickling and change in C (wt.%) before and after sintering and after acid treatment.

	BET Surface Area (m^2^g^−1)^	Average Pore Size (nm)	Pore Volume (cm^3^g^−1^)	C (wt.%)
Before sintering	——	——		7.75
Precursor	7.82	10.16	0.04	4.39
After pickling	12.59	38.68	0.05	0.18

**Table 2:** Adsorption isotherm parameters of the M-T-LIS.

Langmuir			Freundlich		
q_m_ (mg/g)	K_L_ (L/mg)	R^2^	n	K_F_ (L/g)	R^2^
31.68	1.51	0.99	0.01	28.50	0.85

**Table 3:** Kinetics parameters of the M-T-LIS for the adsorption of lithium ions.

Pseudo-First-Order			Pseudo-Second-Order		
q_e1_ (mg/g)	K_1_ (min^−1^)	R^2^	q_e2_ (mg/g)	K_2_ (g/mg/min)	R^2^
4.78	0.01	0.86	31.55	0.57	0.99

**Table 4:** Lithium selectivity from the artificial salt lake brine using the M-T-LIS.

	K^+^	Ca^2+^	Na^+^	Li^+^
*K_d_* (mL/g)	0.07	18.69	0.11	46.47
*α*	674.35	2.49	441.22	1.00

## Data Availability

The authors confirm that the data supporting the findings of this study are available within the article.
